# Impact of Short-Term Fasting on The Rhythmic Expression of the Core Circadian Clock and Clock-Controlled Genes in Skeletal Muscle of Crucian Carp (*Carassius auratus*)

**DOI:** 10.3390/genes9110526

**Published:** 2018-10-29

**Authors:** Ping Wu, Lingsheng Bao, Ruiyong Zhang, Yulong Li, Li Liu, Yuanan Wu, Jianshe Zhang, Zhigang He, Wuying Chu

**Affiliations:** 1 Department of Biological and Environmental Engineering, Changsha University, Changsha 410003, China; wuping@hnu.edu.cn (P.W.); baolingsheng2003@126.com (L.B.); liyulong0801@163.com (Y.L.); jzhang@ccsu.edu.cn (J.Z.); 2 Collaborative Innovation Center for Efficient and Health Production of Fisheries in Hunan Province, Changde 415000, China; 3 Aquatic Biotechnology, University of Duisburg-Essen, 45141 Essen, Germany; ruiyong.zhang@uni-due.de; 4 Fisheries Research Institute of Hunan Province, Changsha 410153, China; hnhhliliu@163.com (L.L.); 15200826136@163.com (Y.W.)

**Keywords:** circadian, rhythms, clock genes, functional genes, fasting, skeletal muscle, crucian carp

## Abstract

The peripheral tissue pacemaker is responsive to light and other zeitgebers, especially food availability. Generally, the pacemaker can be reset and entrained independently of the central circadian structures. Studies involving clock-gene expressional patterns in fish peripheral tissues have attracted considerable attention. However, the rhythmic expression of clock genes in skeletal muscle has only scarcely been investigated. The present study was designed to investigate the core clock and functional gene expression rhythms in crucian carp. Meanwhile, the synchronized effect of food restrictions (short-term fasting) on these rhythms in skeletal muscle was carefully examined. In fed crucian carp, three core clock genes (*Clock*, *Bmal1a*, and *Per1*) and five functional genes (*Epo*, *Fas*, *IGF1R2*, *Jnk1*, and *MyoG*) showed circadian rhythms. By comparison, four core clock genes (*Clock*, *Bmal1a*, *Cry3*, and *Per2*) and six functional genes (*Epo*, *GH*, *IGF2*, *Mstn*, *Pnp5a*, and *Ucp1*) showed circadian rhythms in crucian carp muscle after 7-day fasting. In addition, three core clock genes (*Clock*, *Per1*, and *Per3*) and six functional genes (*Ampk1a*, *Lpl*, *MyoG*, *Pnp5a*, *PPARα*, and *Ucp1*) showed circadian rhythms in crucian carp muscle after 15-day fasting. However, all gene rhythmic expression patterns differed from each other. Furthermore, it was found that the circadian genes could be altered by feed deprivation in crucian carp muscle through the rhythms correlation analysis of the circadian genes and functional genes. Hence, food-anticipatory activity of fish could be adjusted through the food delivery restriction under a light–dark cycle. These results provide a potential application in promoting fish growth by adjusting feeding conditions and nutritional state.

## 1. Introduction

Some aspects of physiology, behavior, and gene expression in fish are regulated by circadian clocks [1,2,3]. The central circadian clock genes have different locations among species. These include pineal gland, retina, and suprachiasmatic nucleus (SCN)-like structure within the hypothalamus in fish [4]. In addition to the central clock components, peripheral tissues of the liver, kidney, heart, and skeletal muscle possess local circadian clocks as well [4,5,6]. Generally, the circadian rhythm of biological activities can be influenced by external stimuli such as feeding and light. For example, in the liver of *Nile tilapia*, the molecular clock of peripheral oscillators can be controlled by a restricted feeding mode [7,8]. Nevertheless, the studies on the effect of fasting on the molecular clock of skeletal muscle in fish are still scarce.

There is widespread agreement that the core components of the molecular clock gene system are formed by two main arms which are positive and negative transcriptional–translational feedback loops [9]. In the positive group, the activators *Clock* and *Bmal1* are known to heterodimerize, thus enhancing promoters transcription of the negative arms encoded by Period (*Per1*, *Per2*, and *Per3*) and Cryptochrome (*Cry1*, *Cry2*, and *Cry3*) genes [10]. When enough PER and CRY complexes are formed and translocated to the nucleus, these complexes inhibit their own transcription and block the function of the *CLOCK:BMAL1* heterodimer. Compared with the core clock genes in mammals, those in fish are more homologous. This theory is confirmed in zebrafish, medaka, goldfish, European seabass, gilthead seabream, Senegalese sole, Atlantic cod, and Chinese perch [11,12,13,14,15,16,17,18].

The aims of this study were twofold: Firstly, to test the daily rhythms of the two positive and six negative loops of the molecular clock genes in crucian carp peripheral (muscle) tissue and, secondly, to explore the effect of short term (7 days and 15 days) starvation on muscle rhythm in crucian carp.

## 2. Materials and Methods

### 2.1. Animals and Tissue Collection

The protocol was approved by the Institutional Animal Care and Use Committee (IACUC) of Changsha University (permit #20128945-1, 2015). All surgeries were performed under sodium pentobarbital or tricaine methanesulfonate (MS-222) anesthesia, and every effort was made to minimize animal suffering. All fish handling procedures conducted during the study were approved by the IACUC Committee.

Before the start of the experiment, crucian carp, with body weight of about 50 ± 5 g, were randomly assigned into three tanks (60 fish per tank); each tank had six divided grid cages (10 fish were randomly placed in each cage). All fish were fed once a day at the same time on a commercial pellet diet (1% body weight, Tongwei Group Co., Ltd., Si Chuan, China) for acclimating ~4 weeks. Fishes were all adapted to a 12L:12D photoperiod, with lights on at zeitgeber time 0 (ZT0). After 1 month, the first cage was used as control for normal feeding during the experiment. The dorsal epaxial muscle samples were collected from six individuals at 6:00, 9:00, 12:00, 15:00, 18:00, 21:00, 24:00, and 3:00 and 6:00 of the following day, which corresponds to ZT0, ZT3, ZT6, ZT9, ZT12, ZT15, ZT18, and ZT21 and ZT24 of the following day. At these points, average fish weight was approximately 50 ± 5 g. The samples were immediately frozen in liquid nitrogen and stored at −80 °C. The remaining two tanks of fish, which experienced unchanged light conditions, were starved for 7 days and 15 days and the same part skeletal muscles were collected as described above, respectively.

### 2.2. RNA Extraction and complementary DNA Synthesis

Total RNA from the muscle tissues was extracted using a Trizol reagent kit (Takara Biotechnology, Dalian, China) following the manufacturer’s standard protocol. Then RNA concentration and purity were investigated by measuring absorbance at 260/280 nm with a Nanodrop 2000 spectrophotometer (Nanodrop Technologies, Wilmington, DE, USA). Meanwhile, 1.5% agarose gel electrophoresis was used to check the integrity and relative quantity of RNA. The Prime Script^TM^ reagent Kit with gDNA Eraser kit (Takara Biotechnology, Dalian, China) was used to synthesize the single strand cDNA.

### 2.3. Quantitative Real-Time PCR (qPCR)

The SYBR^®^ Premix Ex Taq II (Tli RNaseH Plus) (Takara Biotechnology, Dalian, China) was used for quantitative real-time PCR (qPCR) and its amplification reaction was carried out with a Bio-Rad CFX96 system (USA). Primer pairs purchased from Biosune (Shanghai, China) are shown in Table 1. *β*-actin (AB039726.2) was used as an internal control. The total volume of 25 μL reaction mix contained 2 μL cDNA templates, 12.5 μL SYBR Premix Ex Taq, 8.5 μL ddH2O, and 1 μL each of forward and reverse primers (Table 1). The following protocols were used. (1) Predenaturation at 95°C for 30 s; (2) amplification and quantification step, 40 cycles of 95 °C for 5 s, and 58 °C for 25 s; (3) melting curve formation: 65 °C to 95 °C, every rise by 0.5°C to collect fluorescence value at a time. The qPCR analysis of each sample was repeated three times.

### 2.4. Statistical Analysis

The relative expression ratio of target genes was calculated by R = 2^−∆∆*C*t^, where *C*_t_ is the number of cycles when the fluorescence signal reaches a set threshold. All data were analyzed for the tests of normality and equal variance. Normally distributed data were analyzed using ANOVA followed by Ducan post hoc tests using SPSS 16.0 (International Business Machines Corporation, Armonk, NY, USA). Since the distribution of experimental values was bell-shaped, a cyclic Gaussian curve was used as the mathematical model to describe genes rhythms [19,20]. The cosinor analysis was used to evaluate daily rhythmicity in the expression of all the detected genes, cosinor analysis was performed by fitting a periodic sinusoidal function (*f*(*t*) = *M* + *Acos*(*tπ*/12 − *ϕ*) to the expression values of genes across the nine time points, where *f*(*t*) is the gene expression level in a given time, mesor (*M*) is the mean value, *A* is the sinusoidal amplitude of oscillation, *t* is time in hours, and *ϕ* is the acrophase (peak time of the approximating sinusoidal function). Periodic sinusoidal function was performed by MATLAB (Math Works, Natick, MA, USA). The statistical significance *p*-value of cosinor analysis was defined by the noise/signal of amplitude calculated from the ratio *SE*(*A*)/*A*. Expression of these genes was considered to display rhythmicity if it had both *p* < 0.05 by ANOVA and *p*-value < 0.3 by Matlab [16,21]. Correlation of messenger RNA (mRNA) expression levels between the clock genes and functional genes was analyzed using the Pearson’s correlation test (*r*).

## 3. Results

### 3.1. Rhythmic Expression of Core Clock and Functional Genes during a Daily Cycle in the Muscle of Normally Fed Crucian Carp

**Figure 1:**
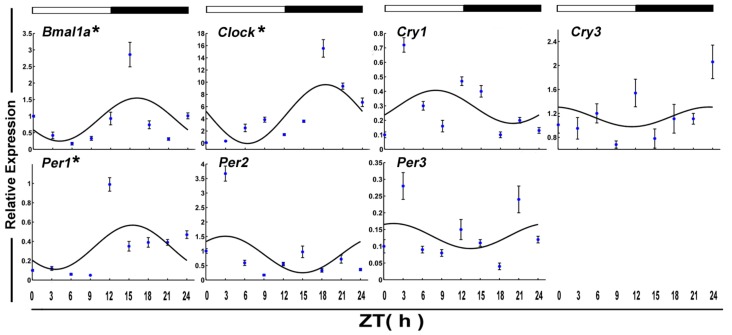
Cosinor analyses of the core clock genes expression levels in the muscle of normally fed crucian carp during the daily light–dark cycle. The values are mean ± SEM (*n* = 6) of the normalized transcript levels of clock genes. White and black, represent the light, dark, and light–dark transition phases, respectively. Expression of these genes was considered to display rhythmicity if it had both *p* < 0.05 by ANOVA and *p*-value < 0.3. An asterisk (*) beside the gene name indicates that the expression is daily rhythmic. ZT: Zeitgeber time.

**Figure 2:**
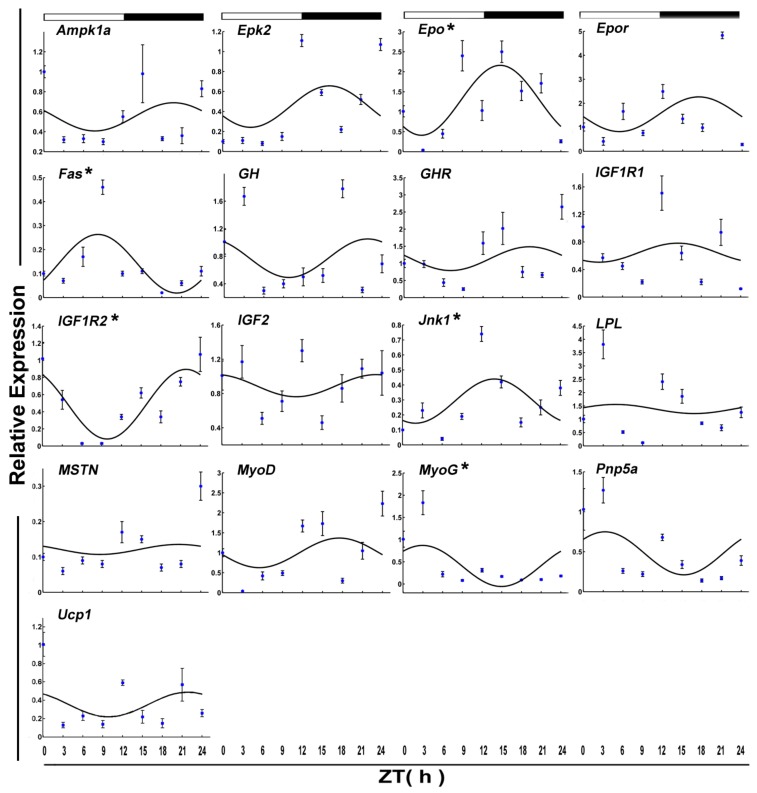
Cosinor analyses of functional genes expression levels in the muscle of the crucian carp fasted for during the daily light–dark cycle. The values are mean ± SEM (*n* = 6) of the normalized transcript levels of each functional genes. White and black, represent the light, dark, and light–dark transition phases, respectively. Expression of these genes was considered to display rhythmicity if it had both *p* < 0.05 by ANOVA and *p*-value < 0.3. An asterisk (*) beside the gene name indicates that the expression is daily rhythmic.

The daily expression profiles of the circadian clock genes shows that the positive arms (*Clock* and *Bmal1a*) and negative arms (*Per1*) displayed a significant daily rhythm in skeletal muscle during the fed state (Figure 1 and Table 2). In addition, the peak expression of *Clock*, *Bmal1a*, and *Per1* occurred at night. Among the functional genes, *Epo*, *Fas*, *Igf1r2*, *Jnk1*, and *MyoG* displayed daily rhythmic expression. Only *Fas* and *MyoG* had an acrophase during the light phase (Figure 2 and Table 2).

### 3.2. Rhythmic Expression of Core Clock and Functional Genes during a Daily Cycle in the Muscle of Crucian Carp after 7-Day Fasting

**Figure 3:**
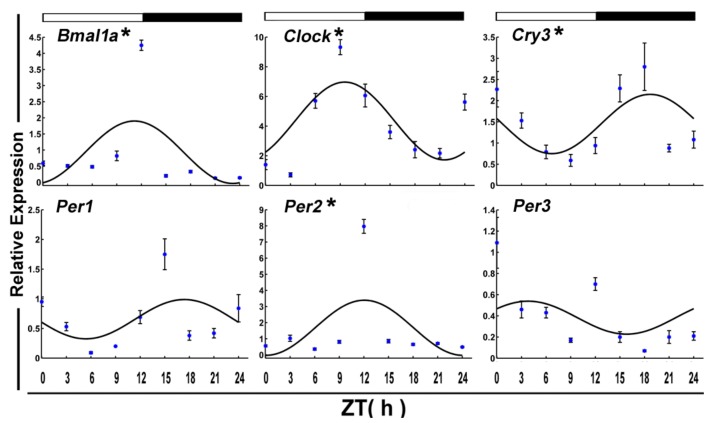
Cosinor analyses of the core clock genes expression levels in the muscle of the crucian carp fasted for one week during the daily light–dark cycle. The values are mean ± SEM (*n* = 9) of the normalized transcript levels of each clock genes. White and black, represent the light, dark, and light–dark transition phases, respectively. Expression of these genes was considered to display rhythmicity if it had both *p* < 0.05 by ANOVA and *p*-value < 0.3. An asterisk (*) beside the gene name indicates that the expression is daily rhythmic.

After 7-day fasting, *Bmal1a* and *Clcok* in crucian carp skeletal muscle still displayed significant daily cyclic oscillations (Figure 3 and Table 2). However, its acrophase occurred at the daytime transition. Meanwhile, fasting disrupted circadian periodicity for *Per1* (*p*-value = 0.37). And the expression of *Cry3* and *Per2* began to show a rhythm. Both of their acrophase reached peak phase at night.

**Figure 4:**
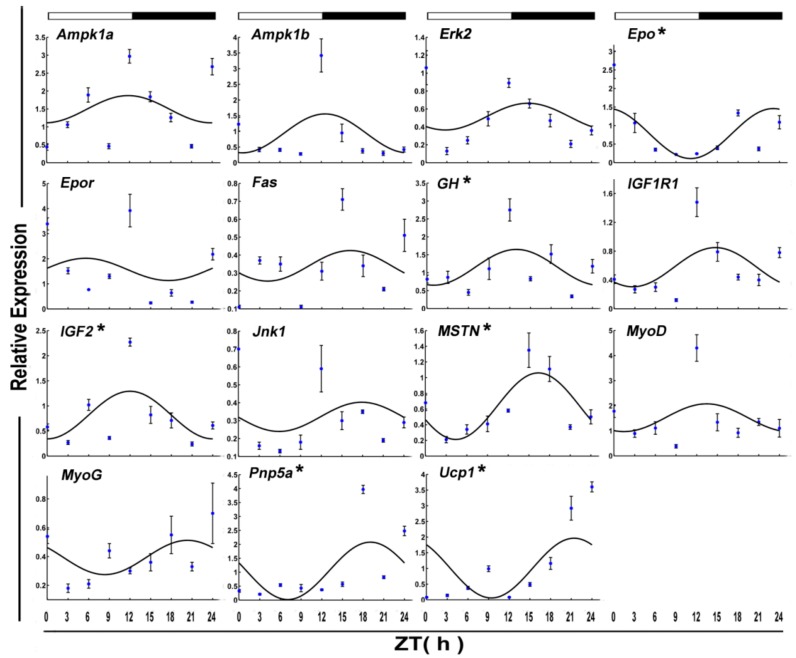
Cosinor analyses of functional genes expression levels in the muscle of the crucian carp fasted for one week during the daily light–dark cycle. The values are mean ± SEM (*n* = 9) of the normalized transcript levels of each functional genes. White and black, represent the light, dark, and light–dark transition phases, respectively. Expression of these genes was considered to display rhythmicity if it had both *p* < 0.05 by ANOVA and *p*-value < 0.3. An asterisk (*) beside the gene name indicates that the expression is daily rhythmic.

The mRNA level of six functional genes in crucian carp skeletal muscle displayed circadian rhythms (Figure 4 and Table 2). Daily rhythmic expression was demonstrated by *Epo* (*p*-value = 0.10), *Gh* (*p*-value = 0.25), *Igf2* (*p*-value = 0.17), *Mstn* (*p*-value = 0.01), *Pnp5a* (*p*-value = 0.20), and *Ucp1* (*p* value = 0.23). But all the rhythmic functional genes (ZT = 8.15) showed an acrophase during the darktime.

### 3.3. Rhythmic Expression of Core Clock and Functional Genes during a Daily Cycle in the Muscle of Crucian Carp after 15-Day Fasting

**Figure 5:**
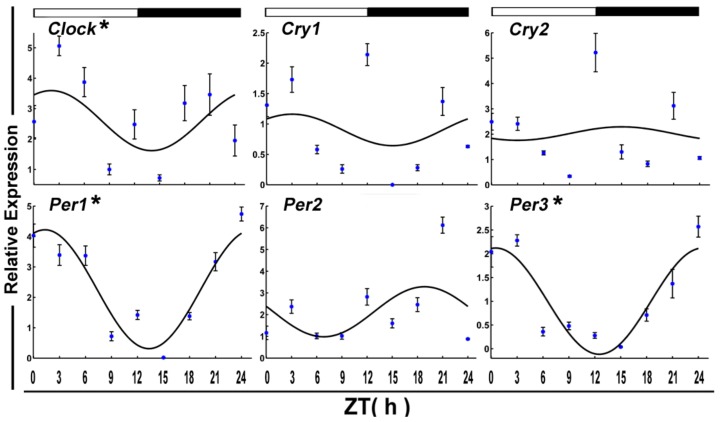
Cosinor analyses of the core clock genes expression levels in the muscle of the crucian carp fasted for 15 days during the daily light–dark cycle. The values are mean ± SEM (*n* = 9) of the normalized transcript levels of each clock genes. White and black, represent the light, dark, and light–dark transition phases, respectively. Expression of these genes was considered to display rhythmicity if it had both *p* < 0.05 by ANOVA and *p*-value < 0.3. An asterisk (*) beside the gene name indicates that the expression is daily rhythmic.

The daily rhythms of eight clock genes transcript levels were examined by qPCR in the skeletal muscle of crucian carp fasted for 15 days during a 12:12 h light/dark cycle (Figure 5). Statistical analysis showed that mRNAs of *Clock*, *Per1*, and *Per3* displayed daily rhythms and all had acrophases in the light phase (ZT = 21.80 h).

After 15 days of starvation, *Ampk1a*, *Lpl MyoG*, *Pnp5a*, *PPARα*, and *Ucp1* expressed in a circadian manner. Among these, two genes, including *Lpl* and *MyoG*, showed a daily rhythm with an acrophase during the dark phase.

### 3.4. Gene Correlation Analysis

The circadian rhythm expression of all genes in fed and fasted crucian carp were analyzed using the correlation test (Table 3). The transcript levels of clock and functional genes displayed intergroup and intragroup positive or negative correlations in these conditions. In the normally fed fish muscle, the components of the transcriptional activator *Clock* and myogenic regulatory factor *MyoG* showed moderate negative correlation (−0.8 < *r* < −0.5). In addition, significant positive correlation was found between *Per1* and transcription level of *Jnk1* (*r* = 0.89).

In the seven-day fasted fish muscle, there were moderate or strong intergroup or intragroup positive and negative correlations in clock genes and functional gene pairs: *Clock:Cry3* (*r* = −0.63), *Clock:Epo* (*r* = −0.58), *Bmal1a*:*Per2* (*r* = 0.98), *Bmal1a*::*GH2* (*r* = 0.86), *Bmal1a:IGF2* (*r* = 0.88), *Cry3*:*Epo* (*r* = 0.62), *Cry3*:*Mstn* (*r* = 0.77), *Per2*:*Gh2* (*r* = 0.87), and *Per2: IGF2* (*r* = 0.88).

**Figure 6:**
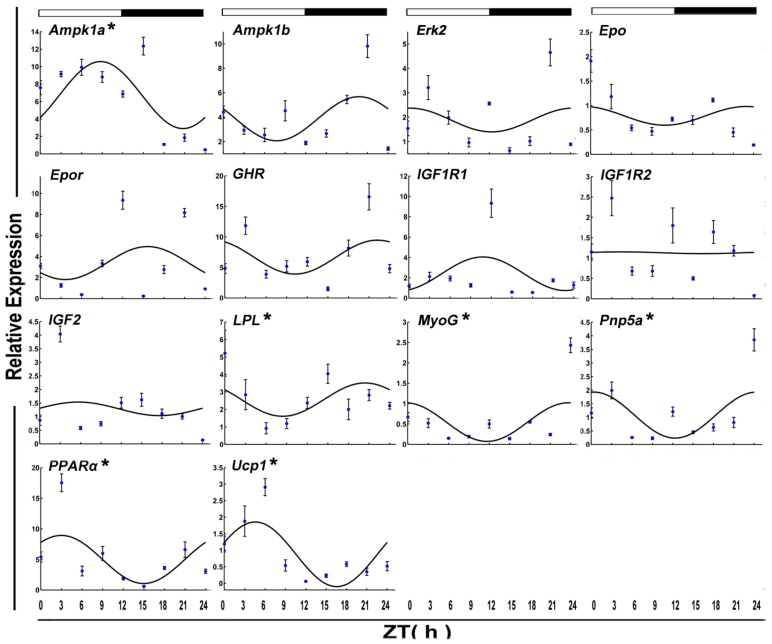
Cosinor analyses of functional genes expression levels in the muscle of the crucian carp fasted for 15 days during the daily light–dark cycle. The values are mean ± SEM (*n* = 9) of the normalized transcript levels of each functional genes. White and black, represent the light, dark, and light–dark transition phases, respectively. Expression of these genes was considered to display rhythmicity if it had both *p* < 0.05 by ANOVA and *p*-value < 0.3. An asterisk (*) beside the gene name indicates that the expression is daily rhythmic.

In the fifteen-day fasted muscle, the daily expression of 12 clock and functional genes showed either positive or negative correlations with each other. Among clock genes, only *Per1* had a strong correlation with *Per3* (*r* > 0.8) (Figure 6). Between the transcripts of rhythmicity clock genes and functional genes, there was also moderate or strong intergroup or intragroup positive and negative correlation. *Per1* displayed moderate positive correlations with *MyoG* and *Pnp5a* (0.5 < *r* < 0.8). Also, *Per3* showed moderate or positive correlations with *MyoG*, *Pnp5a*, and *PPARα*. Interestingly, *Clock*, which had a rhythm again after 15 days of starvation, showed a moderate negative correlation with *Pnp5a* and *Ucp1*.

### 3.5. Effects of Starvation on Circadian Clock and Functional Genes in Crucian Carp

**Figure 7:**
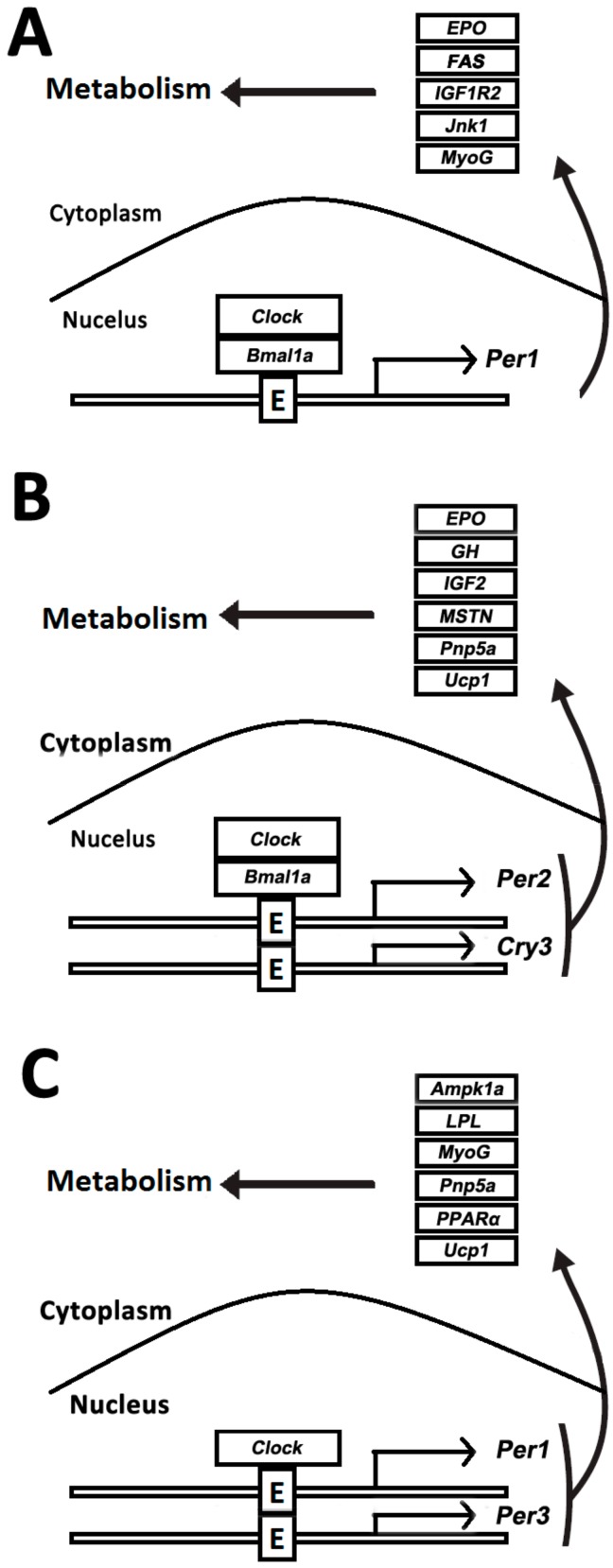
Comparative analysis of rhythmic genes data. (**A**) Rhythmic genes in the muscle of normally fed crucian carp; (**B**) rhythmic genes in the muscle of crucian carp fasted for 7 days; (**C**) rhythmic genes in the muscle of crucian carp fasted for 15 days.

The transcript of circadian clock and functional genes in muscles was comparatively analyzed upon normal feeding and fasting treatment. Apparently, two clock genes, *Clock* and *Bmal1a*, and one functional gene, *Epo*, were rhythmically expressed in both normally fed and 7-day fasting crucian carp skeletal muscle (Figure 7A,B). However, the acrophase of *Clock* and *Bmal1a* exhibited a left shift after 7-day fasting compared with the circadian genes in the normally fed crucian carp. In addition, one clock and four functional genes disappeared and two new clock and five functional genes appeared in the crucian carp muscle during the 7-day fasting treatment.

There was a significant change in gene expression in the normal feeding and starving fish after a longer period of hunger. Two clock genes *Clock* and *Per1*, both showed rhythmic expression pattern in the normally fed and 15-day fasted crucian carp (Figure 7B,C). Compared with the circadian-genes in the normal feeding crucian carp, one clock and four functional genes disappeared and one clock and five functional genes appeared in the crucian carp muscle after 15-day starvation. Obviously, the circadian profile in crucian carp muscle was altered upon food deprivation, namely, starvation.

**Figure 8:**
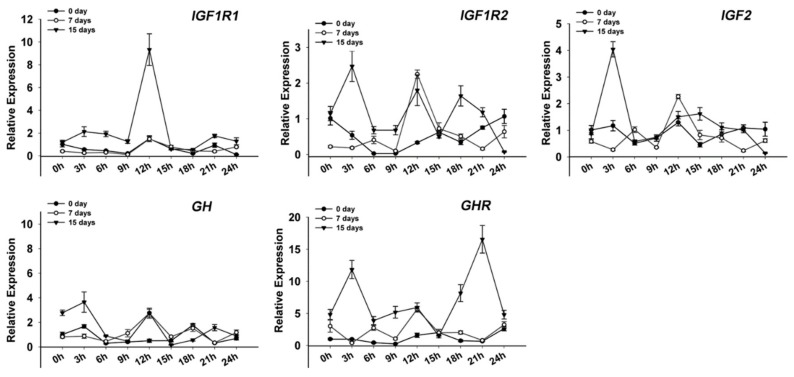
Expression of five growth hormone/insulin-like growth factor (GH/IGF) axis genes (GH, GHR, IGF1R1, IGF1R2, and IGF2) in the crucian carp muscle during fed and starvation. The values are expressed as the mean ± SEM (*n* = 9) of the normalized transcript levels of each GH/IGF axis genes.

Two functional genes (*Pnp5a* and *Ucp1*) were circadian after 7-day and 15-day fasting treatments (Figure 7A,C). However, three clock and four functional genes disappeared and two new clock and four functional genes appeared after 15-day fasting. It is interesting that only the *Clock* gene remained rhythmic during starvation. Meanwhile, the results showed that the expression of five genes (*GH*, *GHR*, *IGF1R1*, *IGF1R2*, and *IGF2*), which serve as growth hormone regulation (*GH*) /insulin-like growth factor *(IGF*) axis, increased to constant levels in the fasted fish (Figure 8).

## 4. Discussion

This is the first report on the rhythmic expression of the circadian clock and functional genes in crucian carp skeletal muscle. We detected the rhythm of the eight core clock genes, namely two members of the transcriptional activator (*Clock* and *Bmal1a*) and six genes of the transcriptional repressor (*Cry1*, *Cry2*, *Cry3*, *Per1*, *Per2*, and *Per3*). We also tested the rhythm of 22 functional genes involved in sugar and lipid metabolism in normal feeding and fasting state.

In earlier reports on the Chinese perch, we identified a series of circadian clock and myogenic genes that may participate in controlling muscle biological clock system [17]. Few reports are available on changes in rhythmic expression of fish clock genes after starvation. In this study, we analyzed the positive factor *Clock* expression in crucian carp and showed that it maintained rhythm briefly after short-term of starvation. When the fish was starved longer its rhythm was recovered and a shift of acrophase was observed in the fasted group. This discovery is consistent with the report that the rhythmic expression of *Clock* in tilapia liver: expression changed with different feeding conditions [8]. In this study, *Clock* expression was also strongly associated with negative regulatory factor *Per1* in the normally fed state. The above phenomena were also found in zebrafish and mammals [7,11]. Another positive element, *Bmal1a*, expressed a peak at the dark phase, which has been also observed in Atlantic cod skeletal muscle [16]. This is opposite to previous reports that *Bmal1a* expression showed an acrophase at the end of the light phase and the beginning of the dark phase at the normally fed condition in zebrafish, medaka, gilthead seabream, tilapia, and rainbow trout brain [8,22,23,24,25]. In addition, *Bmal1* could not only participate in maintaining the circadian oscillators, but also contribute to lipid metabolism control in mammals [26,27]. In Atlantic cod liver, *PPARα* can combine with the *Bmal1* promoter and form stable cyclic oscillations with the rhythmic expression of *Bmal1* [28]. While in crucian carp skeletal muscle, this phenomenon has also been found. Only after 15 days of starvation *PPARα* began to be rhythmical. These suggest that the biological clock system of muscle is functioning very different from the central molecular oscillators, especially after fasting.

Previous studies of the peripheral of the vertebrate’s circadian oscillators revealed the presence of the transcription activator *Clock-Bmal1*, which drives the rhythmic expression of the repressor *Per* and *Cry*. Similarly, *Per* and *Cry* play an important role in photo transduction and circadian photosensitivity. This is confirmed in *Drosophila*, zebrafish, and mice [6,22,29]. Because of the existence of multiple copies of *Per* and *Cry* genes, the negative regulatory gene system of fish is more complex. With the change of nutrient conditions, the rhythm of the negative regulatory genes became more unstable and changeable, e.g., *Per1* showed rhythmicity in normally fed and 15-day fasting conditions with different amplitude. It is noteworthy that *Per1* was the only gene that had rhythms during the normally fed condition in crucian carp skeletal muscle. These patterns are similar to those reported for other *Per1* genes in the zebrafish, goldfish, Chinese perch, and European sea bass [11,17,21,30]. After 7-day fasting, the daily rhythmic expression of *Per2* took the place of *Per1* and had a strong correlation with *Bmal1a*. But after longer starvation, *Per1* showed a positive correlation with *Per3*. These results suggested that *Per* may interact with *Cry* to control the transcriptional activation and function in the circadian feedback loop. Thus, we speculated that the correlation between the negative regulatory factors *Per* and *Cry* was unstable. However, there was a positive regulatory relationship between the negative (*Per* and *Cry*) and positive regulators (*Clock-Bmal1*).

Most of the functional genes studied displayed temporal changes in transcript levels during the daily cycle and fasting treatment in mice muscle. As an important factor in promoting muscle growth, *Igfs* and *Ghs* have been studied and reported earlier [31,32]. In zebrafish, *igf1ra*, *igf1rb*, *igfbp1a*, and *igfbp1b* were upregulated during fasting and two *Igf*-binding proteins genes (*Igfbp3* and *Igfbp5b*) were rhythmic and were conformed involvement of the clock pathway [11]. In the present study, the expression of three *Igfs* and two *Ghs* increased during the process of starvation. Even in the feeding cycle, rhythmic *Igf1r2* was consistent with the expression pattern of *Igfbp3* and *Igfbp5b* of zebrafish which had an acrophase during the dark time. In the 7 days of starvation, *Bmal1a* had a strong correlation with rhythmic *Gh2* and *Igf2*. This confirmed that they were involved in the regulation of the biological clock. After 15 days of starvation, there were no *Igfs* and *Ghs* displayed significant cyclic oscillations. A plausible hypothesis is that a long-time starvation causes the fish no longer growing, and (*Gh*)/(*Igfs*) axis only maintains basic metabolism and no longer participates in the regulation of the biological clock.

The interaction between the ligands and *Igf1*-receptors ultimately leads to muscle growth; this also affects the expression of some genes related to myogenesis. As the member of the myogenic regulatory factors (*Mrfs*), *MyoD* and *MyoG* are classes of helix-loop-helix transcription factors that play a vital role in myogenesis [33]. In mice, the two polymers formed by *Clock* and *Bmal* directly act on the distal regulatory region (DRR) of the *MyoD* promoter. Thus, *MyoD* acts as a molecular link between the circadian clock system and skeletal muscle physiological activity [34]. Simultaneously, evidence has been found for circadian expression of *MyoD* in Chinese perch and Atlantic cod [16,17]. In contrast, we found that *MyoD* did not show rhythm in crucian carp skeletal muscle no matter in normal feeding or starvation. This discovery was consistent with the report in zebrafish [11]. But the expression of *MyoG* exhibited a circadian expression pattern peaking in phase with *Clock* in daily cycle during the light time and in phase with *Bmal1a* after 15 days of fasting during the dark period (Table 3). It could be suggested that the rhythmic expression of *MyoG* in crucian carp muscle parallels that described for *MyoD* in mouse, Chinese perch, and Atlantic cod muscle, with potential effects on the maintenance of myofibrillar structure.

This study addresses, for the first time, the activities of key functional genes related to glycolipid metabolism and their possible cross-regulation of expression of genes related to myogenesis during normal feeding and starvation in skeletal muscle. These data advance our understanding of the complex muscle regulation of circadian clock and metabolism response to feeding. This information, as an additional biological clock control system beyond the central oscillator, has potential to regulate the severity of circadian rhythm disruption and metabolic disorders.

## 5. Conclusions

This study investigated the impact of restricting feeding on the expression of the core circadian clock and functional genes in crucian carp skeletal muscle. The results confirmed that among the eight core clock and 22 functional genes analyzed, three core clock genes and six functional genes had daily rhythms in the muscle of normal fed crucian carp. Three core clock genes and eight functional genes showed circadian rhythms after 7-day and 15-day fasting, but the gene and rhythmic expression patterns were different. Rhythms and correlation analysis of the circadian genes and functional genes indicates that feed deprivation could alter the circadian gene components in crucian carp muscle. Therefore, this study provides a potential application on fish growth by managing feeding conditions and nutritional state.

## Figures and Tables

**Table 1:** Primer sequences used in the study.

Gene	Forward Primer (5′-3′)	Reverse Primer (5′-3′)	Tm (°C)
*Clock*	CTCATTGGTCATCTGCCGTC	GGTGGTTCTTTCGGGTCAAT	62
*Bmal1a*	AAGCCAGCATTCTTGTCGGA	AAACCGACTCCGAGACGAAC	62
*Cry1*	AGTCGCTTGTTTCCTCACCC	ACAGCCACATCCAGCTTCCT	57
*Cry2*	CCAACCCCCGATTTGATAAG	TGAGAAAACAAGCGACAGCG	62
*Cry3*	TCAATCACTGTTCGCAAGCC	AAAACTCTCGCCACAGCAGC	62
*Per1*	GAATGAGCACCAGCAAAGCG	CGGGAATCAATGAAGACCTG	62
*Per2*	CCGCAAAGTTTCCTTCGTCA	ATCCTCTTCCTCTTGTCGCA	62
*Per3*	GCAGCCTCTACAAGAAGCCC	GCCGCTGTGGGTTTGTCTTC	62
*Ampk1a*	CTCAGCGGTAGATTACTGCCACA	ACATTTTCAGGCTTGAGGTCTCT	62
*Ampk1b*	CACCCCTCCGTTATTAGCCT	CTTCTCGCACACCTCCTTCA	62
*EPO*	ATTACGCCCCATCTGTGACC	GACGCCTGTAATGAGCCGAT	62
*EPOR*	GATACGCAGCGGAGGGAAGT	GCGTGACTCCAAACACAGGC	63
ERK2	CCACAGAGACCTGAAGCCAT	CCAGAATACAACCCACCGAC	60
*Fas*	CCCCGAAGAAATGGAAAACT	TTGCTGCCACGCATAGACAC	62
*GH*	TGAAAATGGGCATCAGTGTG	TGAAGCAAGCCAGCAGACGA	62
*GHR*	CACACAGCAGTCCATCTACGG	TCACCACTCCAATCATTCCAA	62
*IGF1R1*	GAACCACAAAAACCCAACGG	CCGCACACGGGCAGAATAGT	63
*IGF1R2*	AGGACAAGCACATTCTGGGG	TGCCAACGGAGCAGGTAGAG	58
*IGF2*	GAGTGCTGCTTTCGGAGTTG	TGGATGGGACCCCTCTTCTT	62
*JNK1*	CTGGAGCACAAGGCATCGTC	GGGTTTGGTTCTGGAAGGGT	60
*LPL*	GATGGACGGTCACGGGTATG	GTGTAGGGTAGTGCTGTTGCG	62
*MyoD*	CACACAGCAGTCCATCTACGG	TCACCACTCCAATCATTCCAA	63
*MyoG*	TTTTTACGAAGGCGGCGATA	AGTGCTGCTGCTCCTGGTGA	62
*MSTN*	CGGCTGGGACTGGATTATTG	GGAGACATCTTGGTGGGGGT	56
*Pnp5a*	GCAGGGACGGTTTCATCTCTA	TGTTTCCAGCAAATCCAGGCA	62
*PPARα*	AATGCCACAGTCGGAGAAGC	GGAGGTGTGCTCGTCTTGCC	62
*Ucp1*	CTGCCCAACATCACGAGGAA	CGAACGCAGACACGAAATGA	54
*β-actin*	CCGTGACATCAAGGAGAAGC	GGAAGGATGGCTGGAAAAGA	58

**Table 2:** Parameters defining the gene expression rhythms in skeletal muscle of crucian carp (*Carassius auratus*).

Gene	Fed					Fasted-7d					Fasted-15d				
	Amplitude	Mesor	Acrophase (h)	*p*-value	*p*	Amplitude	Mesor	Acrophase (h)	*p*-value	*p*	Amplitude	Mesor	Acrophase (h)	*p*-value	*p*
***Clock***	**4.82**	**4.78**	**18.43**	**0.08**	**>0.05**	**2.62**	**4.34**	**9.54**	**0.06**	**>0.05**	**0.99**	**2.60**	**2.03**	**0.24**	**>0.05**
***Bmal1a***	**0.65**	**0.90**	**16.08**	**0.20**	**>0.05**	**0.97**	**0.93**	**11.15**	**0.20**	**>0.05**	-	-	-	-	<0.05
***Cry1***	0.11	0.29	7.95	0.51	>0.05	-	-	-	-	<0.05	0.26	0.90	3.04	0.76	>0.05
***Cry2***	-	-	-	-	<0.05	-	-	-	-	<0.05	0.27	2.02	15.01	0.93	>0.05
***Cry3***	0.16	1.14	23.45	0.68	>0.05	**0.31**	**0.64**	**18.69**	**0.14**	**>0.05**	-	-	-	-	<0.05
***Per1***	**0.23**	**0.34**	**15.55**	**0.22**	**>0.05**	0.33	0.66	17.38	0.37	>0.05	**1.95**	**2.27**	**1.30**	**0.00**	**>0.05**
***Per2***	0.63	0.88	2.98	0.42	>0.05	**1.71**	**1.68**	**12.07**	**0.24**	**>0.05**	1.15	2.13	18.82	0.32	>0.05
***Per3***	0.04	0.13	1.49	0.55	>0.05	0.16	0.38	3.82	0.60	>0.05	**1.12**	**1.00**	**3.99**	**0.00**	**>0.05**
***Ampk1a***	0.14	0.55	19.72	0.61	>0.05	0.38	1.49	11.80	0.67	>0.05	**3.84**	**6.74**	**8.77**	**0.10**	**>0.05**
***Ampk1b***	-	-	-	-	<0.05	0.62	0.94	12.57	0.35	>0.05	1.80	3.87	19.75	0.30	>0.05
***Erk2***	0.21	0.45	16.23	0.57	>0.05	0.15	0.51	14.78	0.58	>0.05	0.49	1.88	0.34	0.71	>0.05
***EPO***	**0.87**	**1.29**	**14.77**	**0.04**	**>0.05**	**0.68**	**0.78**	**23.11**	**0.10**	**>0.05**	0.19	0.79	22.92	0.71	>0.05
***EPOR***	0.72	1.54	17.44	0.57	>0.05	0.44	1.58	5.58	0.80	>0.05	1.57	3.38	15.62	0.60	>0.05
***FAS***	**0.12**	**0.14**	**8.29**	**0.07**	**>0.05**	0.09	0.34	16.12	0.64	>0.05	-	-	-	-	<0.05
***GH***	0.28	0.77	21.81	0.55	>0.05	**0.50**	**1.15**	**12.95**	**0.25**	**>0.05**	-	-	-	-	<0.05
***GHR***	0.35	1.14	19.06	0.66	>0.05	-	-	-	-	<0.05	2.77	6.99	22.19	0.38	>0.05
***IGF1R1***	0.14	0.64	14.44	0.81	>0.05	0.27	0.58	14.67	0.32	>0.05	1.65	2.40	10.77	0.37	>0.05
***IGF1R2***	**0.41**	**0.49**	**21.83**	**0.01**	**>0.05**	-	-	-	-	<0.05	0.02	1.13	4.32	1.00	>0.05
***IGF2***	0.13	0.89	23.15	0.60	>0.05	**0.47**	**0.82**	**11.99**	**0.17**	**>0.05**	0.25	1.29	5.65	0.91	>0.05
***Jnk1***	**0.15**	**0.29**	**13.91**	**0.26**	**>0.05**	0.08	0.32	16.95	0.70	>0.05	-	-	-	-	<0.05
***LPL***	0.17	1.39	4.85	0.96	>0.05	-	-	-	-	<0.05	**0.95**	**2.56**	**20.41**	**0.28**	**>0.05**
***Mstn***	0.01	0.12	20.64	0.93	>0.05	**0.42**	**0.64**	**16.39**	**0.01**	**>0.05**	-	-	-	-	<0.05
***MyoD***	0.37	1.00	17.51	0.59	>0.05	0.55	1.52	13.43	0.54	>0.05	-	-	-	-	<0.05
***MyoG***	**0.46**	**0.41**	**2.94**	**0.19**	**>0.05**	0.12	0.39	20.37	0.30	>0.05	**0.47**	**0.55**	**23.65**	**0.29**	**>0.05**
***Pnp5a***	0.27	0.48	3.27	0.33	>0.05	**1.03**	**1.05**	**19.06**	**0.20**	**>0.05**	**0.85**	**1.08**	**0.33**	**0.20**	**>0.05**
***PPARa***	-	-	-	-	<0.05	-	-	-	-	<0.05	**3.93**	**4.99**	**2.98**	**0.18**	**>0.05**
***Ucp1***	0.13	0.35	21.84	0.61	>0.05	**0.95**	**1.01**	**21.40**	**0.23**	**>0.05**	**0.98**	**0.88**	**4.55**	**0.03**	**>0.05**

Note: Amplitude, half the distance between peaks of the fitted wave form; Mesor, the average cycle value; Acrophase, time point in the cycle of highest amplitude; The cosinor analysis was used to obtain the rhythmic parameters as defined by a sinusoidal function. Statistical significance was assumed when the ratio *p*-value was below 0.3 (in bold).

**Table 3:** The correlation of rhythmic clock and functional genes in skeletal muscle of crucian carp (*Carassius auratus*).

Fed		Fasted-7d		Fasted-15d	
Gene Pairs	*r*	Gene Pairs	*r*	Gene Pairs	*r*
*Clock:MyoG*	−0.55	*Clock:Cry3*	−0.63	*Clock:Per1*	0.52
*Per1:Jnk1*	0.89	*Bmal1a: Per2*	0.98	*Per1:Per3*	0.83
		*Clock:EPO*	−0.58	*Clock:Pnp5a*	0.67
		*Bmal1a: GH*	0.86	*Clock:Ucp1*	0.63
		*Bmal1a:IGF2*	0.88	*Per1:MyoG*	0.60
		*Cry3:EPO*	0.62	*Per1: Pnp5a*	0.65
		*Cry3: MSTN*	0.77	*Per3:MyoG*	0.68
		*Per2:GH*	0.87	*Per3: Pnp5a*	0.79
		*Per2: IGF2*	0.88	*Per3: Ppara*	0.56

Note: only correlations with *r* > 0.50 or *r* < −0.50 are shown. The values were set to define the degree of correlation: data are moderately correlated if 0.50 < *r* < 0.80 or −0.80 < *r* < −0.50 and there is a strong correlation when *r* ≥ 0.80 or *r* ≤ −0.80.
